# Fidelity of the representation of value in decision-making

**DOI:** 10.1371/journal.pcbi.1005405

**Published:** 2017-03-01

**Authors:** Paul M. Bays, Ben A. Dowding

**Affiliations:** University of Cambridge, Department of Psychology, Cambridge, United Kingdom; Oxford University, UNITED KINGDOM

## Abstract

The ability to make optimal decisions depends on evaluating the expected rewards associated with different potential actions. This process is critically dependent on the fidelity with which reward value information can be maintained in the nervous system. Here we directly probe the fidelity of value representation following a standard reinforcement learning task. The results demonstrate a previously-unrecognized bias in the representation of value: extreme reward values, both low and high, are stored significantly more accurately and precisely than intermediate rewards. The symmetry between low and high rewards pertained despite substantially higher frequency of exposure to high rewards, resulting from preferential exploitation of more rewarding options. The observed variation in fidelity of value representation retrospectively predicted performance on the reinforcement learning task, demonstrating that the bias in representation has an impact on decision-making. A second experiment in which one or other extreme-valued option was omitted from the learning sequence showed that representational fidelity is primarily determined by the relative position of an encoded value on the scale of rewards experienced during learning. Both variability and guessing decreased with the reduction in the number of options, consistent with allocation of a limited representational resource. These findings have implications for existing models of reward-based learning, which typically assume defectless representation of reward value.

## Author summary

Many models of learning and decision-making assume that experienced rewards are stored without error. We examined this assumption experimentally: participants first learned an association between different options and rewards in a simple two-alternative choice task. We then asked them to report what reward they expected to receive for each of the options they had experienced. We checked that the reports we collected matched performance on the choice task, meaning that the values participants reported were the same as those they used to decide between options. The results showed that participants were both less precise (greater variability) and less accurate (greater bias) in their reports of middling reward values compared to either high- or low-valued options. Reports of high and low values were similar in quality even though participants had experienced the rewards associated with high-value options considerably more often. Whether an option’s value was stored well or poorly was not fixed, but instead depended on how the value compared to other options the participant had experienced. These results should lead to better models of how decisions are made based on experiences of reward.

## Introduction

In an uncertain and dynamic environment, rational decision-making depends on the ability to learn, store and update the reward values associated with different choices or actions [1, 2]. This ability in turn depends on the coding of reward in neurons of the prefrontal cortex [3–8], supported by teaching signals carried by projections from the basal ganglia [9–13]. Like neurons throughout the brain [14], the firing of reward-sensitive neurons is stochastic, i.e. noisy. However, little is known about how this noise is expressed in the representation of reward value.

Classical learning algorithms [4, 15–17] describe how the values associated with different options are updated, and the decision rules that determine what choices are taken. These models typically assume that values are stored flawlessly: suboptimal decisions are instead a result of noise in reward-generating processes (making experience of past rewards an imprecise guide to the future), incomplete updating of reward estimates by new information (as parameterized by a learning rate), or stochastic decision rules such as *ϵ*-greedy or softmax. While these models can provide good approximations to observed learning patterns, they could be improved by more accurately reflecting what is inevitably an imperfect representation of value in the nervous system.

A second class of decision models, based on noisy accumulation of evidence [18–20], have been shown to account for features of deliberation time as well as a number of violations of rational choice exhibited by human decision-making. In these models, decisions are generated by leaky integration of value information with random variability in each update step. A key assumption of these models is that the noise component is constant across different magnitudes of reward: this assumption has not previously been tested.

Here, we assessed the fidelity of value representation by first running participants on a typical reinforcement learning task in which they were trained to associate different options with particular reward magnitudes. At the end of the learning session, participants were subject to a surprise test in which they were required to directly report the reward they expected to receive on choosing each of the previously-experienced options. Because each participant was able to provide only a single estimate for each learned action-reward pair, a large number of participants were required to obtain interpretable response distributions; for this reason we ran the experiments using a crowdsourcing service.

## Results

**Fig 1:**
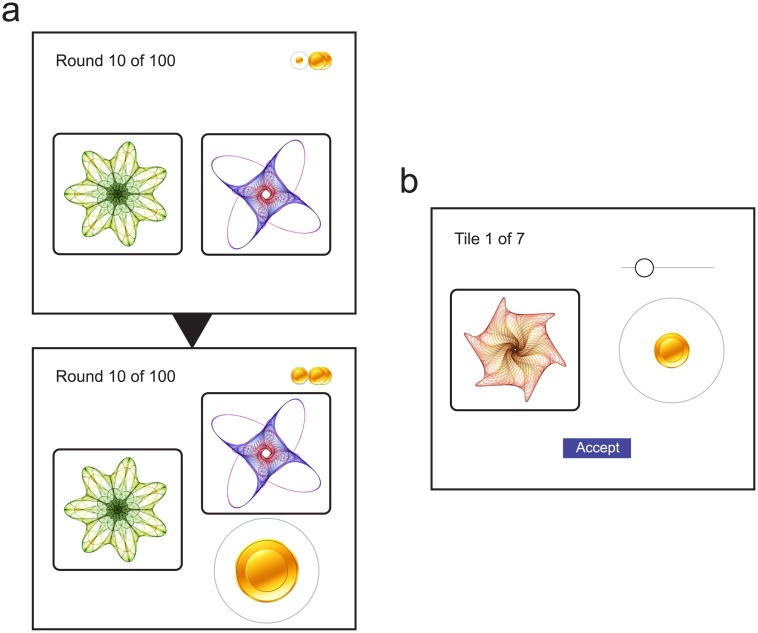
Experimental task. (a) During the learning session, participants chose from pairs of options represented by fractal tiles and were presented with rewards represented by coins that varied in size. (b) During an unexpected testing session, participants were instructed to report the expected reward associated with each tile by dragging a slider to change the size of a coin.

**Fig 2:**
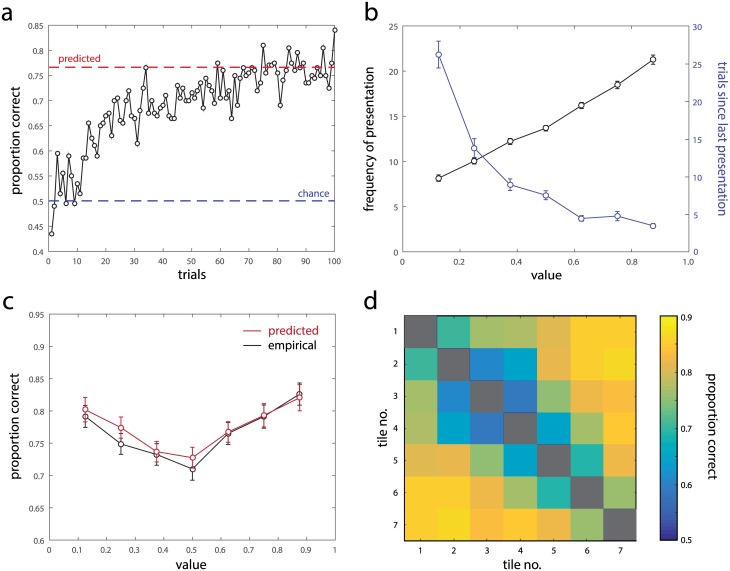
Results from the learning session. (a) Proportion of trials on which the higher-valued tile was chosen, as a function of trial number. Blue dashed line indicates chance performance. Red dashed line indicates the proportion predicted based on reports in the subsequent testing session. (b) Frequency with which tiles of each value were revealed during the learning session (black), and number of trials elapsed since the last presentation of a tile value at the end of the session (blue). (c) Proportion of trials (black symbols) on which the higher-valued tile was chosen, as a function of presented tile value, at the end of the session (final 25 trials). Every trial on which a tile of a specific value was presented as an option is included in each data point, therefore each trial contributes to two datapoints. Red symbols indicate the proportion predicted based on data from the testing session. (d) Proportion of trials on which the higher-valued tile was chosen, for each of the possible pairs of tiles (ordered by increasing value).

Participants completed a reinforcement learning task (Exp 1; Fig 1a) in which they selected from pairs of options, represented by fractal image tiles, and received rewards corresponding to the value of the chosen tile plus random noise. Over the course of 100 trials, participants learned associations between the tiles and expected rewards: the frequency with which the option with higher mean value was chosen increased from chance (50%) to reach a plateau at approximately 75% (Fig 2a).

Because participants observed rewards associated only with tiles they selected, there was substantial variation in the frequency with which different tile values were presented (Fig 2b). The reward associated with the highest-valued tile was presented almost three times as frequently as the lowest-valued (8.2 ± 0.4 vs 21.3 ± 0.5; M ± SE), and at the end of the learning session on average more than seven times as many trials had elapsed since the last selection of the lowest-valued tile compared to the highest-valued (26.2 ± 1.8 vs 3.5 ± 0.3).

Despite these strong differences in the frequency and recency with which rewards were presented, by the end of the learning session the probability of choosing the correct tile varied only weakly between trials involving the highest- and lowest-valued tiles (Fig 2c, black symbols; mean difference between symmetrically-valued pairs of tiles [e.g. 0.875 vs 0.125]: 3.7% ± 1.5%). Instead, we observed that probability correct followed an approximately U-shaped function of value, with trials involving the extreme-valued tiles substantially more likely to be correct than those involving intermediate values (mean difference between extreme- and middle-valued tiles: 9.8% ± 1.7%).

While the superior performance for trials involving extreme-valued options could reflect differences in the representational fidelity of extreme versus intermediate values, it could also be artifactual, arising because trials involving an extreme-valued option on average have a larger disparity in value between the two options presented. To address this, we examined probability correct for pairs of tiles separated by the minimum relative value difference of 0.125. We again found that performance was significantly better for extreme-valued than intermediate-valued tiles (p < 0.03), confirming that these performance differences are not due to differences in value disparity (no significant effects were observed for larger relative value differences, p > 0.16; probability correct for each tile pair is shown in Fig 2d).

**Fig 3:**
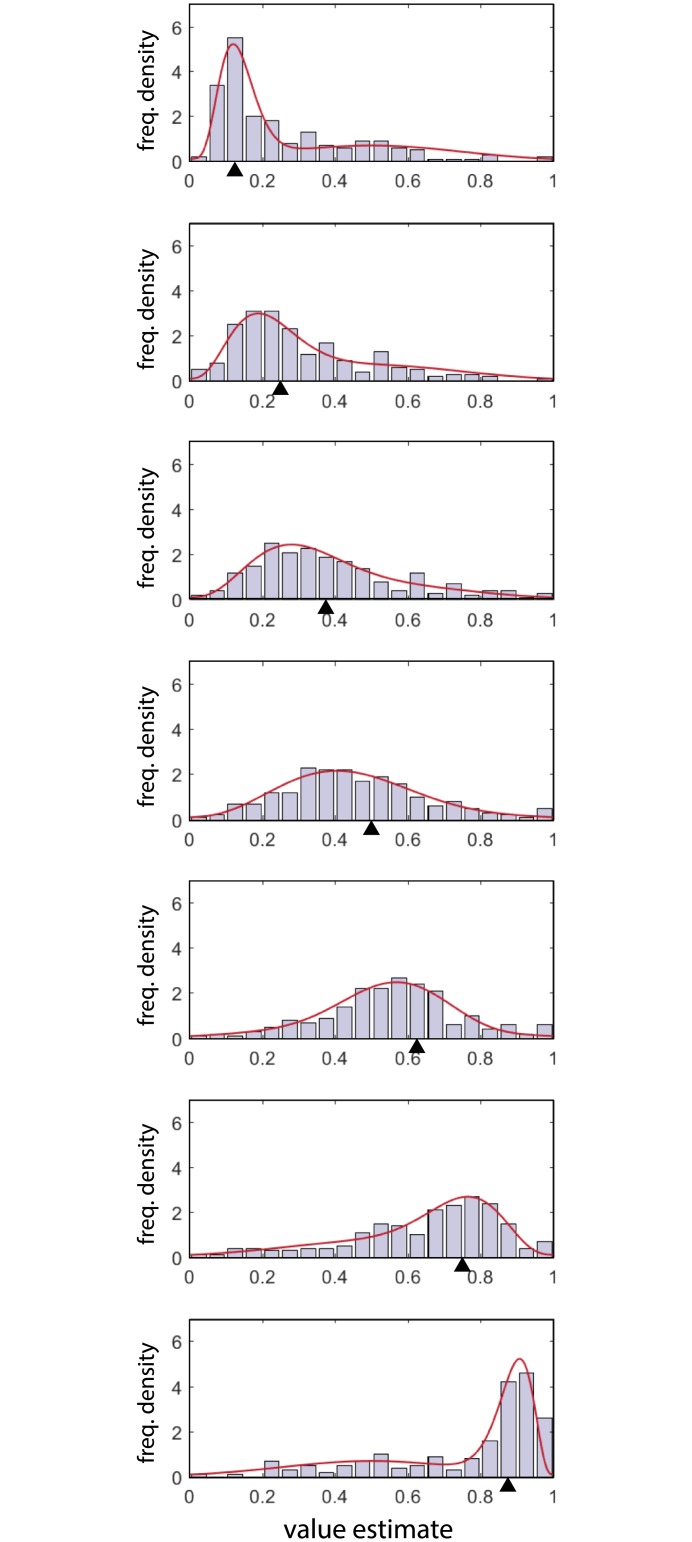
Value estimates. Bars indicate distributions of value estimates reported in the testing session, for true tile values indicated by arrows (increasing top to bottom). Red curves are maximum likelihood fits of the mixture model illustrated in Fig 4.

The learning session was followed by a surprise testing session (Fig 1b), in which participants were required to report the value they associated with each of the options they had been presented with during learning. Fig 3 (grey bars) plots the distributions of response estimates for each mean reward value. Note that, because of the random variability in presented item values, the mean observed value of a tile during the learning session could differ from the tile’s expected value. However, these deviations were very small (mean absolute deviation < 0.01) compared to the observed variability in reproduction (mean absolute error 0.16), indicating that internal noise was by far the dominant factor in determining response variability. We therefore do not consider these deviations further.

Before we draw inferences on the basis of these distributions, we would like to ensure that they reflect the true variability in the representations of value used by participants to make their decisions in the learning task. This is particularly pertinant as the distributions are obtained across, rather than within, participants, with each participant contributing a single sample to each of the histograms in Fig 3. We therefore calculated the frequency with which participants would choose correctly on each trial if their internal representations of value matched their reports in the testing session (see Methods).

The red dashed line in Fig 2a shows the mean predicted proportion correct calculated on this basis. This value (76.6% ± 1.3%) was highly consistent with empirically-observed performance at the end of the learning session (last 25 trials: 76.4% ± 1.4%, p = 0.46). Red symbols in Fig 2c plot the predicted proportion correct as a function of tile value: empirical frequencies obtained over the last 25 learning trials were statistically indistinguishable from the predictions based on reported values in the testing session (all p > 0.16). We conclude that the distributions of reported value estimates over participants accurately correspond to the actual value information used by participants in decision-making.

Consistent with results from the learning session, we found only very weak correlations between error on the report task and the frequency or recency with which a reward was presented during learning (frequency, mean *r* = −0.087, p = 0.002; recency, mean *r* = 0.058, p = 0.042).

**Fig 4:**
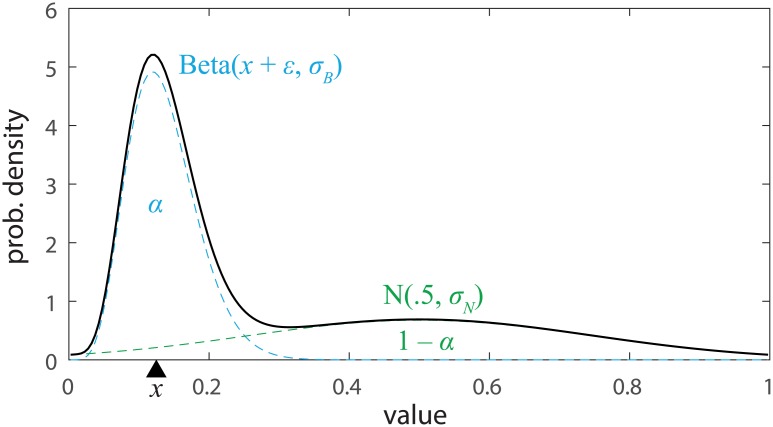
Mixture model. The model of value estimates consisted of a mixture of a beta distribution (blue), corresponding to imprecise recall of the target value, and a normal distribution (green), capturing guessing.

To capture the key properties of the distributions shown in Fig 3 we fit them with a mixture of two component distributions. One, corresponding to an imprecise report of the true value of a tile, was represented by a beta distribution centered on the true value with some bias (the beta distribution is a bell-shaped distribution similar to the normal but confined to the range 0–1); the other, corresponding to random guessing, by a normal distribution centered in the middle of the value range (we used a normal rather than a uniform distribution to capture any bias in guesses towards the center of the range; in the absence of such a bias, the normal component could approximate a uniform distribution to arbitrary exactness). The mixture model is illustrated in Fig 4.

We tested three different mixture models, differing in whether the width of the beta distribution, the mixture proportion, or both could vary across different tile values. In the best fitting model, the mixture proportion was fixed, indicating that the probability of guessing did not vary with tile value; however the width of the beta distribution did vary, indicating that there were differences in how precisely the different options were represented (AICc: width-only −840.5, both −836.2, mixture-only −715.5; BIC: width-only −757.0, both −721.5, mixture-only −632.0). The fits of the best fitting model are plotted in red in Fig 3.

**Fig 5:**
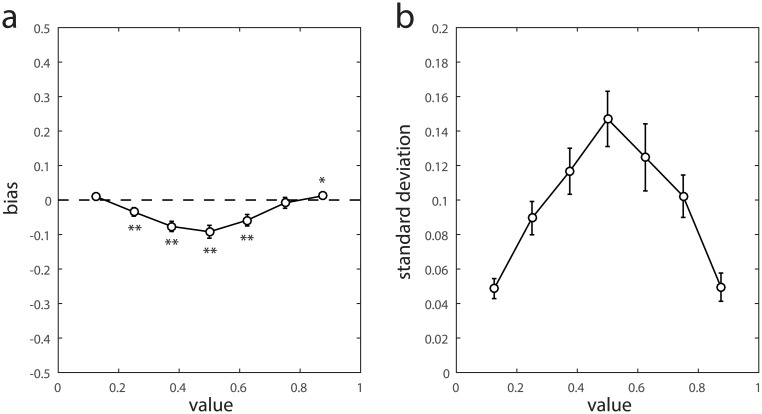
Maximum likelihood model parameters. (a) Bias of the fitted beta distribution mean relative to the true tile value. Asterisks indicate significant deviation from zero (* p < 0.05; ** p < 0.01). (b) Standard deviation of the fitted beta distribution.

Fig 5a & 5b plot the bias and variability, respectively, of the beta component of the best-fitting model as a function of option value. We observed significant biases towards lower values for intermediate reward values only (asterisks in Fig 5a indicate significance; we also observed a very small but statistically significant bias towards higher values for the highest-valued tile). Symmetrically-valued pairs of tiles (i.e. those on opposite sides of the middle tile value) had similar biases (0.875 vs 0.125: difference = 0.002, p > 0.05; 0.75 vs 0.25: difference = 0.026, p < 0.05; 0.625 vs 0.375: difference = 0.016, p > 0.05).

Variability also depended strongly on tile value (Fig 5b): the standard deviation of responses around the correct tile value was approximately three times higher for the middle-valued tiles than for either of the extreme-valued tiles (0.15 vs {0.049, 0.050}, p < 0.01). There were no significant differences in variability between symmetrically-valued pairs of tiles (all p > 0.05).

An additional analysis confined to only those participants (89 in total) who demonstrated a strongly significant (p < 0.01) correlation between estimated and true tile values, revealed very similar magnitudes of effect of tile value on bias and variability, indicating that these effects were not limited to observers with poor overall recall.

These results indicate that extreme-valued options are represented more precisely and with less bias than intermediate-valued options. This effect could reflect either the relative value of an option within the range of values experienced, or it could reflect the absolute value relative to the bounds on possible responses in the testing session. To disambiguate these two possibilities we ran a second experiment (Exp 2) in which only six tiles were presented (one fewer than in Exp 1). The excluded tile was either the lowest- or the highest-valued tile from Exp 1.

**Fig 6:**
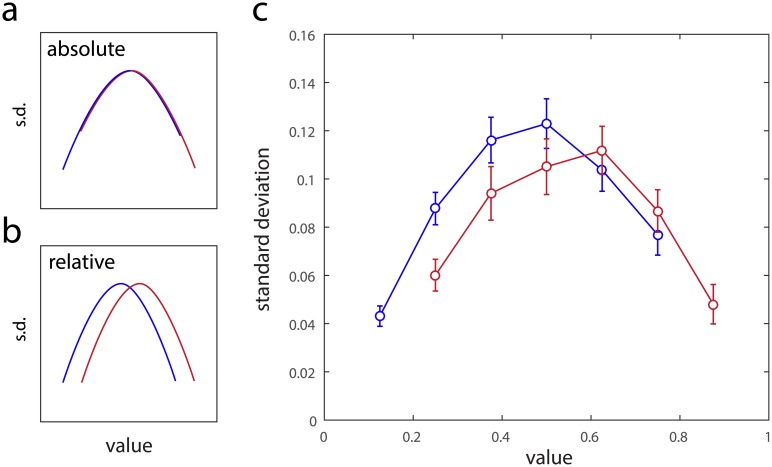
Absolute versus relative coding affecting variability. (a) Predicted variability for a model in which variability is determined by the absolute value within the bounds [0, 1]. Blue curve indicates participants for whom the highest-valued tile was omitted, red the lowest-valued. The model predicts that the two curves will exactly overlap. (b) Predicted variability for a model in which variability is determined by the relative value within the range of all rewards experienced during learning. The model predicts that the two curves will be translated relative to each other. (c) Empirical standard deviations obtained in Exp 2.

Fig 6a & 6b illustrate the predictions of the two models for representational variability. If variability is determined by a tile value’s absolute position within the range of possible responses, the standard deviations of responses of participants who experienced all but the lowest-valued tile (Fig 6a, red) should exactly overlie those of participants who experienced all but the highest-valued tile (blue). In contrast, if variability is determined by the relative position within the range of experienced tile values, the two curves should be displaced along the x-axis by a difference of one tile value (Fig 6b). Fig 6c plots the observed data. The two curves do not overlap (significant difference at tile value 0.25, p < 0.05), however a formal model comparison could not consistently distinguish between the two possibilities (AICc difference [relative − absolute]: 8.8 ± 15.9, p > 0.05; BIC difference: 4.9 ± 15.9, p > 0.05).

**Fig 7:**
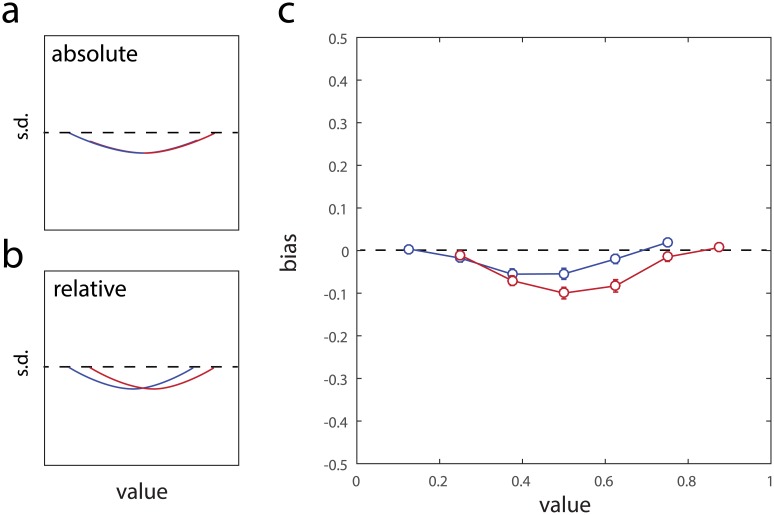
Absolute versus relative coding affecting bias. (a) Predicted bias for a model in which bias is determined by the absolute value within the bounds [0, 1]. Blue curve indicates participants for whom the highest-valued tile was omitted, red the lowest-valued. The model predicts that the two curves will exactly overlap. (b) Predicted bias for a model in which bias is determined by the relative value within the range of all rewards experienced during learning. The model predicts that the two curves will be translated relative to each other. (c) Empirical biases obtained in Exp 2.

Fig 7 presents results of an identical analysis for representational bias. Here, model comparison found strong evidence in favour of a relative coding of value representations (AICc difference [relative − absolute]: −43.2 ± 20.3, p < 0.05; BIC difference: −47.1 ± 20.3, p < 0.05)

As in Exp 1, we found minimal correlation between error on the report task and the frequency or recency of reward (frequency, mean *r* = −0.040, p = 0.071; recency, mean *r* = 0.042, p = 0.067).

Comparing the distributions of value estimates between the experiments with six and seven tiles revealed an overall increase in mean variability of the beta component with increasing number of tiles (*σ*_*B*_ = 0.088 vs 0.097, p < 0.05) and an increase in guessing (*α* = 0.67 vs 0.58, p < 0.05). There was no significant effect on the mean bias of the beta component (*ε* = −0.033 vs −0.043, p > 0.05) nor the width of the normal (guessing) component (*σ*_*N*_ = 0.25 vs 0.26, p > 0.05).

## Discussion

We examined the nature of internal representation of reward by following a standard reinforcement learning task with an unexpected test, in which participants directly reported the rewards they associated with previously-experienced choices. The results demonstrated a substantial advantage in the fidelity of representation for extreme values: both low and high value rewards were represented with lower variability and less bias than intermediate values. These differences in fidelity mapped onto the decisions participants made during learning, retrospectively predicting how accurately participants chose between the different options.

In our interactions with the world, we preferentially exploit options that we associate with the largest rewards. For this reason, our experience unequally samples the distribution of available rewards, favoring higher values. This effect was apparent in our reinforcement learning task: although the lowest and highest rewards were made available on equal numbers of trials, the frequency with which participants were exposed to the highest rewards was many times that of the lowest. Remarkably, this oversampling of high rewards had negligible impact on the fidelity with which reward values were maintained: the lowest values were represented with the same bias and variability as the highest. This effect on action-reward associations differs dramatically from that observed for memory of other stimuli, e.g. word lists, where the accuracy with which associations are recalled depends strongly on both the frequency and recency of presentation [21, 22]. A future study could test the effects of presenting both chosen and unchosen tile values on each trial: given the absence of frequency and recency effects in the present study, we predict that this would have minimal impact on response fidelity.

Theoretically, differences in the fidelity of reported value representations could be determined by a reward’s relative position in the range of experienced values, or they could depend on the reward’s absolute position on the scale of permitted responses. I.e., the more accurate reproduction of the highest reward values could arise because the reward is the highest of those experienced during learning, or because the reward is close to the edge of the response range. The absolute-coding hypothesis makes the prediction that, if one of the extreme values in the learning task is omitted, fidelity of the remaining values will not depend on which value was omitted, as this does not change their position on the absolute scale of responses. The relative-coding hypothesis makes the opposite prediction, because omitting an extreme value changes the relative position of the other values on the scale of experienced values. We performed this experiment: a model comparison based on report variability did not clearly disambiguate the two hypotheses, suggesting both may play a role, whereas a model comparison based on report bias strongly favoured the relative-coding hypothesis.

An additional consideration also supports the relative-coding hypothesis: the distributions of value estimates obtained by direct report very accurately reproduced performance on the learning task, indicating that the fidelity of reproduction faithfully reflected fidelity of the representations used to make choices in the preceding task. Critically, at the time these choices were made, participants had no knowledge or experience of the report task. This strongly argues against any specific effect of the response space in generating our results. We conclude that it is the value relative to the range of values experienced during learning that most strongly determines fidelity. This finding is consistent with observations of context-dependence in decision-making [23, 24] and relative coding in neural representations of value [25–27], which may be a consequence of divisive normalization [28].

While we have focused on the fidelity with which value is represented, a recent study [29] has obtained converging results by examining the salience of reward memories. This study presented participants with options that led with equal frequency to an extreme or an intermediate reward. On a subsequent memory test, participants were asked to report which outcome came most readily to mind when presented with each option in turn. The experimenters observed a strong bias towards reporting the extreme value over the intermediate value associated with each option. Participants also overestimated the frequency with which the extreme value was awarded in comparison with the intermediate value.

Recent advances in our understanding of working memory have focused on the concept of a limited memory resource that determines how precisely information is maintained [30]. Two observations have led to this characterization: first, the fidelity of representation of simple visual elements, such as orientations, declines monotonically with increasing number of elements in memory [31–33]; second, differences in the salience or goal relevance of elements results in enhanced fidelity for high priority elements and a consequent decrease in fidelity for those of lower priority [34–36]. The present results pertaining to the internal representation of value may be best understood within a similar framework. Thus, during learning, information regarding action-reward associations accumulates, increasing the fidelity of representation until an upper bound is reached resulting from a resource limit. The fidelity with which reward values are maintained at this limit may be determined by their motivational salience, favoring accurate representation of the lowest and highest values over motivationally-neutral intermediate values.

If fidelity is determined by a limit on available representational resources, rather than limited experience with each action-reward pair, this would account for the absence of frequency and recency effects. Further evidence consistent with a resource account comes from a comparison between learning with six and seven different action-reward pairs. The fidelity of value reproduction for all pairs was enhanced when the total number reduced, as a consequence of a decrease in both variability and guessing. Although the two conditions also differed in the frequency and recency with which the different rewards were presented, the very weak correlations between these factors and response error suggest they are unlikely to have contributed substantially to the difference between conditions. Nonetheless, the present study was not designed to test a resource hypothesis for reward representation, and this proposal, and the link to working memory, remain speculative at this time.

Consideration of how value is represented in cortical spiking activity provides an alternative to the motivational-salience account of the representational advantage for extreme values. In prefrontal cortex, reward-coding neurons display two opposing patterns of activity: roughly half of neurons increase their firing linearly with increasing value, whereas the other half decrease their firing [37, 38]. Spiking activity in cortex is approximately Poisson, i.e. standard deviation increases with the square root of the mean: hence higher firing rates encode information with greater fidelity [39]. We propose that, in such an opponent-coding system, the highest and lowest values, which elicit maximum firing in half the neural population, can be decoded with greater precision than intermediate values, which produce an intermediate firing rate in the whole population. While this would provide a parsimonious explanation for the differences in precision observed in our experiments, it should be noted that opponent-coding schemes are not universal in the brain: neither subcortical nor parietal reward-sensitive neurons display inverse relationships between firing rate and value [12, 40].

With respect to the increased underestimation bias observed for intermediate values, we speculate this may have a Bayesian explanation: greater uncertainty in the internal representation of these values leads to a greater bias towards prior expectations. This would imply that participants’ prior belief is that individual actions will result in small rewards. This could be a fixed prior, similar to the low-velocity prior evident in perception [41], or it could depend on details of how a participant’s expectations are set up by the instructions and study design.

One caveat to our conclusions is that they are based on learning of action-reward associations on a short timescale, on the order of tens of minutes. While this is typical of laboratory studies of human reinforcement learning (e.g. [4, 42–44]), under ecological conditions we often make decisions based on associations learned over much longer periods, even years. Future research will examine whether the observations on fidelity presented here extend to longer timescales of learning. Another consideration is that we have examined only the representation of positive rewards; future work could investigate the fidelity with which behavioral costs are represented. Based on the success of the relative-coding hypothesis (Exp 2), we predict that if the range of experienced option values extended from negative to positive, fidelity would increase with absolute value; however, this will need to be confirmed by future experiments.

We found that the distribution of reported reward values was well-described by a mixture of two-components: one centered on the target value with some bias and variability, the other independent of the target but having some bias towards the center of the value range. While we have described the latter distribution as due to “guessing”, it may not be the case that these responses are purely random. Based on findings in the psychophysics and working memory literature, it is probable that some of these responses actually reflect so-called “swap” errors, in which a participant incorrectly responds with the value corresponding to a tile other than the one they are cued to report (e.g. [45, 46]). Assessments of the frequency of guessing will also depend on the choice of distribution for the non-guessing component: we chose a beta distribution as it is a normal-like distribution in common use with support on the range zero to one. In conjunction with a normally-distributed guessing component, this distribution proved qualitatively to be a good fit to data, however we do not rule out the possibility that the true distribution of “on-target” responses differs from the beta, e.g. by virtue of being long-tailed [47].

Established models of reinforcement learning [15–17] do not typically consider the possibility of bias or variability in value representation. Where noise enters into the models at all it is typically at the decision stage, for example as a softmax decision rule [4]. In contrast, the present results suggest that a major contribution to the stochasticity in decision-making is due to variability in the internal representation of value, rather than in its evaluation. Taking into account the fidelity of reward representation, and in particular the biases favoring extreme values, will be critical for developing a fuller understanding of reward-based learning.

## Methods

### Ethics statement

All participants gave informed consent, in accordance with the Declaration of Helsinki. The study was approved by the Cambridge Psychology Research Ethics Committee.

### Participants

Six hundred participants were recruited and run using Amazon Mechanical Turk (https://www.mturk.com). They were paid $0.50 for their time plus a bonus determined by rewards accumulated during the task (typically in the range $0.50 to $1). Participants completed the experimental tasks on their own computers or laptops; touchscreen devices were automatically excluded. Participants completed a demographic survey, reporting their sex, age, location, education, current illnesses and any vision problems. Twenty-six participants were subsequently excluded from analysis because they reported problems with their vision, or their age fell outside the range 18–60.

### Experiment 1

Two hundred participants took part in Exp 1. The experiment was divided into two parts: in the learning session, participants made choices between pairs of options (“tiles”) and received rewards. In the subsequent testing session, participants reported the reward value they associated with each option. The learning session was introduced by a short tutorial which did not mention the existence of the testing session.

The learning session consisted of 100 trials. On each trial, two tiles (fractal images) were presented (Fig 1a, top) and the participant selected one with a mouse click. The selected tile moved to reveal a reward (Fig 1a, bottom), represented by a coin: the diameter of the coin indicated the reward value, with larger coins corresponding to more reward. Participants were instructed to collect as much reward as possible, which would be converted into a bonus payment at the end of the experiment. A running total of the reward accumulated so far was present at all times in the upper-right of the screen.

The two tiles presented on each trial were selected randomly without replacement from a set of seven. Each tile was associated with a different mean reward value, evenly-spaced in the range 0.125–0.875, where a reward of 0 was indicated by no coin and a reward of 1 was indicated by the largest coin. The actual reward value obtained on each trial was drawn from a beta distribution with mean corresponding to the selected tile’s value and standard deviation 0.035. The assignment of fractal images to mean reward values was randomized for each participant.

In the testing session, which followed immediately after the end of the learning session, each of the seven tiles used in the preceding session was presented one at a time (Fig 1b) and participants were instructed to report the reward they expected to receive for choosing that tile, by dragging a slider which changed the size of a coin. Once they were satisfied that they had adjusted the coin size to match the expected reward they clicked a button marked “accept”.

After the testing session, participants were presented with feedback of the correct reward values associated with each tile, and told how much bonus they had earned. Participants could take as long as they wanted over each part of the experiment, but the whole task typically took about 15 minutes to complete.

### Experiment 2

Exp 2 was identical to Exp 1, except that only six tiles were used. The mean reward values for the six tiles were chosen by excluding either the lowest (Exp 2a; 200 participants) or highest (Exp 2b; 200 participants) value tile from the seven tiles used in Exp 1.

### Analysis

We defined a learning trial as correct if the tile chosen was the one with the higher mean value. To assess whether the distribution of value estimates obtained in the testing session matched performance on the learning task, we calculated for each subject the performance we would expect if their choices were based on their reported value estimates, i.e. if the response for each possible pair of tile values was determined by which tile had the higher value estimate in the testing session. These predicted frequencies were compared to the actual frequencies of correct trials at the end of the learning session (final 25 trials).

The distribution of value estimates $\hat{x}$ obtained across participants in the testing session for each mean tile value *x* was fit with a mixture of a beta distribution centered on the true mean value with bias *ε* and standard deviation *σ*_*B*_, and a normal distribution (intended to capture guessing) centered in the middle of the range of values (0.5) with standard deviation *σ*_*N*_. The mixture parameter *α* corresponded to the proportion of the beta distribution in the mixture. Formally,

$$p\left(\hat{x}\right)=\alpha \beta \left(\hat{x};x+\varepsilon ,\sigma_{B}\right)+\left(1-\alpha \right)\phi \left(\hat{x};0.5,\sigma_{N}\right),$$ (1)

where *β*(*x*; *μ*, *σ*) is the probability density function of the beta distribution with mean *μ* and standard deviation *σ*, and *ϕ*(*x*; *μ*, *σ*) is the probability density function of the normal distribution with mean *μ* and standard deviation *σ*. Note that the beta distribution is commonly parameterized by two shape parameters, *a* and *b*: these can be obtained from the mean and standard deviation as *a* = (*μ*^2^ − *μ*^3^)/*σ*^2^ − *μ* and *b* = *a*(1/*μ* − 1).

Three variants of the model described by Eq 1 were tested. In one, the standard deviation of the beta component *σ*_*B*_ was allowed to vary with the mean tile value, while a single value of the mixture parameter *α* was used for all tile values. In the second, a single value of *σ*_*B*_ was used but *α* was allowed to vary. In the third, both *σ*_*B*_ and *α* varied with mean tile value. In all cases, the bias parameter *ϵ* was allowed to vary with mean tile value.

Maximum likelihood model parameters were obtained by the Nelder-Mead simplex method (*fminsearch* in MATLAB). Models were compared using the Akaike Information Criterion with a correction for finite sample sizes (AICc; [48]) and the Bayesian Information Criterion (BIC). Standard errors and confidence intervals on model parameters and differences between model parameters were calculated by bootstrapping: 1000 resampled datasets were generated by random sampling with replacement from the original dataset, and models fit to the resampled data to obtain a sampling distribution of parameters. Statistically significant differences between model parameters were reported when the bootstrap 95% confidence interval did not encompass zero.
